# Synergistic killing of human small cell lung cancer cells by the Bcl-2-inositol 1,4,5-trisphosphate receptor disruptor BIRD-2 and the BH3-mimetic ABT-263

**DOI:** 10.1038/cddis.2015.355

**Published:** 2015-12-31

**Authors:** E F Greenberg, K S McColl, F Zhong, G Wildey, A Dowlati, C W Distelhorst

**Affiliations:** 1 Division of Hematology/Oncology, Department of Medicine, Case Western Reserve University School of Medicine and University Hospitals Case Medical Center, Cleveland, OH, USA; 2 Department of Medicine, MetroHealth Medical Center, Cleveland, OH, USA; 3 Case Comprehensive Cancer Center, Cleveland, OH, USA

## Abstract

Small cell lung cancer (SCLC) has an annual mortality approaching that of breast and prostate cancer. Although sensitive to initial chemotherapy, SCLC rapidly develops resistance, leading to less effective second-line therapies. SCLC cells often overexpress Bcl-2, which protects cells from apoptosis both by sequestering pro-apoptotic family members and by modulating inositol 1,4,5-trisphosphate receptor (IP_3_R)-mediated calcium signaling. BH3-mimetic agents such as ABT-263 disrupt the former activity but have limited activity in SCLC patients. Here we report for the first time that Bcl-2-IP_3_ receptor disruptor-2 (BIRD-2), a decoy peptide that binds to the BH4 domain of Bcl-2 and prevents Bcl-2 interaction with IP_3_Rs, induces cell death in a wide range of SCLC lines, including ABT-263-resistant lines. BIRD-2-induced death of SCLC cells appears to be a form of caspase-independent apoptosis mediated by calpain activation. By targeting different regions of the Bcl-2 protein and different mechanisms of action, BIRD-2 and ABT-263 induce cell death synergistically. Based on these findings, we propose that targeting the Bcl-2–IP_3_R interaction be pursued as a novel therapeutic strategy for SCLC, either by developing BIRD-2 itself as a therapeutic agent or by developing small-molecule inhibitors that mimic BIRD-2.

Lung cancer accounts for 12% of all new cancers worldwide and is a leading cause of cancer-related mortality in the United States.^1, 2, 3^ Although small cell lung cancer (SCLC) comprises only 15% of lung cancer cases,^2, 3^ it has an annual mortality rate approaching that of breast and prostate cancer.^4^ Compared with the more common non-small cell lung cancer (NSCLC), SCLC is more aggressive and associated with rapid development of metastasis.^2^ Moreover, although SCLC is more responsive to chemotherapy and radiation therapy initially, it typically relapses quickly with treatment-resistant disease.^2^ In contrast to dramatic advances in chemotherapy and personalized medicine in other malignancies, the life expectancy of SCLC patients has remained <2 years for decades and is <1 year for patients with extensive disease.^5, 6^ The lethality of SCLC is attributed in part to the development of resistance to standard combination chemotherapies, underscoring the need to develop novel therapeutic approaches based on understanding the molecular and cellular biology of SCLC.^5, 6^

Evasion from apoptosis is a major hallmark of cancer and a prominent factor underlying drug resistance in SCLC.^3^ Multiple mechanisms contribute to apoptosis resistance in SCLC, including elevated expression of the antiapoptotic Bcl-2 protein^3^ (Supplementary Figure S1). Tsujimoto and colleagues discovered elevated levels of Bcl-2 mRNA and protein in SCLC cells not long after their identification of Bcl-2 as the protein product of the *bcl-2* gene in follicular lymphoma.^7, 8^ Subsequently, immunohistochemistry of 164 primary SCLC samples revealed 76% were positive for Bcl-2, a finding substantiated by microarray detection of increased *BCL-2* mRNA levels in 84% of SCLC samples^9, 10^ and by genomic sequencing of circulating SCLC tumor cells.^11^ Moreover, proteomic profiling documented that Bcl-2 is more highly expressed in SCLC than in NSCLC, reflecting the vastly different biology of these lung cancer subtypes.^12^

The major known function of Bcl-2 is to bind and sequester BH3-only proteins such as Bim, preventing these proteins from inducing apoptosis.^13, 14, 15^ Therefore, a major investment has been made in targeting this interaction for cancer treatment. The interaction takes place in a hydrophobic groove on Bcl-2 and the therapeutic strategy for targeting this interaction has been to develop small molecules, BH3-mimetic agents, which bind in the hydrophobic groove and induce apoptosis by displacing the BH3-only proteins. This approach has been reviewed in detail.^14, 15, 16^

Among BH3-mimetic agents advancing through clinical trials for both hematological malignancies^15, 17^ and solid tumors^18^ are ABT-737 and its orally bioavailable derivative ABT-263 (Navitoclax). Reported studies of ABT-199, a selective inhibitor of Bcl-2, are at present limited to hematological malignancies.^18^ In screening a large number of cancer cell lines, the pioneering work of Oltersdorf *et al.*^19^ demonstrated potent single-agent activity of ABT-737 against cell lines representative of lymphoid malignancies and SCLC. Clinical trials of ABT-263, an orally bioavailable version of ABT-737, achieved overall response rates ranging from as high as 35% in relapsed/refractory chronic lymphocytic leukemia (CLL) and 22% in follicular lymphoma.^17^ Reported responses are generally less in solid tumors with the notable exception of SCLC.^18^ But even in SCLC, activity of ABT-263 is limited in comparison to hematological malignancies, with 1 of the 39 (3%) of patients achieving a partial response to ABT-263 and 9 of the 37 (23%) achieving stable disease in a phase I clinical trial.^20^ This experience suggests a need to develop additional ways of targeting Bcl-2 for cancer treatment.

A potential alternative therapeutic target for Bcl-2-positive malignancies involves interaction of Bcl-2 with the inositol 1,4,5-trisphosphate receptor (IP_3_R), an IP_3_-gated Ca^2+^ channel located on the endoplasmic reticulum (ER). Bcl-2 is located not only on the outer mitochondrial membrane but also on the ER, and at both of these locations, it functions as a potent inhibitor of apoptosis.^21, 22, 23^ ER-localized Bcl-2 interacts with IP_3_Rs and inhibits apoptosis by preventing excessive IP_3_R-mediated Ca^2+^ transfer from the ER lumen into the cytoplasm and nearby mitochondria.^24, 25, 26^ Notably, regions of Bcl-2 involved in binding BH3-only proteins and IP_3_Rs are entirely different. Whereas BH3-only proteins and their BH3-mimetic counterparts bind in a hydrophobic groove composed of BH3 domains 1–3 of Bcl-2,^13, 14^ the BH4 domain of Bcl-2 is necessary for interaction with IP_3_Rs.^27^ To develop a peptide inhibitor of Bcl-2–IP_3_R interaction, we identified the Bcl-2-binding region on the IP_3_R and developed a small synthetic 20 amino-acid peptide corresponding to this region.^28^ This peptide, when fused to the cell-penetrating peptide of HIV TAT, binds to the BH4 domain of Bcl-2 and functions as a decoy peptide, inhibiting Bcl-2–IP_3_R interaction.^29, 30^ We currently refer to this peptide as BIRD-2 (**B**cl-2-**I**P_3_ **R**eceptor **D**isruptor-**2**), having formerly named it TAT-IDP_DD/AA_.^31^ By disrupting the Bcl-2–IP_3_R interaction, BIRD-2 abrogates Bcl-2 control over IP_3_R-mediated Ca^2+^ elevation and induces Ca^2+^-mediated apoptosis in primary human CLL cells^29^ and diffuse large B-cell lymphoma cells.^32^ Notably, BIRD-2 does not kill normal cells, including human lymphocytes isolated from peripheral blood^29^ and normal murine embryonic fibroblasts (F Zhong and C Distelhorst, unpublished data).

The present investigation was undertaken to determine whether Bcl-2–IP_3_R interaction is a potentially useful therapeutic target in SCLC. In support of this concept, we find the majority of SCLC lines tested are sensitive to BIRD-2-induced apoptosis and that BIRD-2 induces apoptosis in several ABT-263-resistant SCLC lines. BIRD-2, we find, lacks generalized cytotoxicity as it does not induce cell death in NSCLC lines or a normal lung epithelial line. On the other hand, we find that BIRD-2 and ABT-263 synergize in killing SCLC cells. These findings for the first time provide preclinical evidence of the potential value of targeting both antiapoptotic mechanisms of Bcl-2 for the treatment of SCLC.

## Results

### BIRD-2-induced cell death

**Figure 1:**
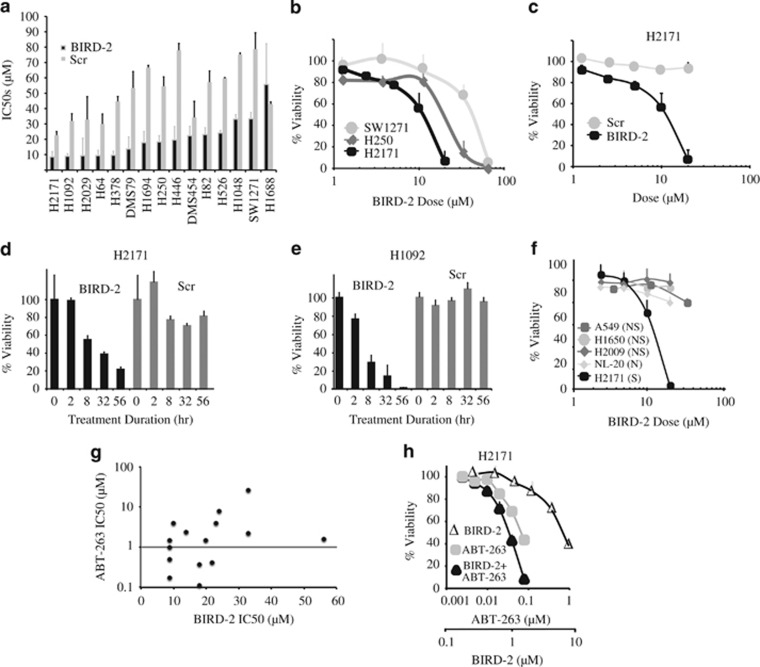
Cell death induction by BIRD-2. (**a**) IC50s of BIRD-2 and Scr control peptide (scrambled version of BIRD-2) in SCLC lines treated for 48 h with a broad range of peptide concentrations. Full dose–response curves were repeated at least three times. (**b**) Representative dose–response curves of SCLC lines treated for 48 h with BIRD-2. (**c**) Representative dose–response curves comparing H2171 cells treated for 48 h with BIRD-2 or Scr. (**d** and **e**) Time course of cell death induction by BIRD-2 in SCLC lines treated with 15 *μ*M (H2171) or 20 *μ*M (H1092) BIRD-2 or Scr at corresponding concentrations. (**f**) Dose–response curves comparing BIRD-2 sensitivity in SCLC cells (S), NSCLC cells (NS), and normal lung epithelial cells (N), each treated for 24 h with BIRD-2 at the indicated concentrations. (**g**) Graph based on data in Supplementary Table S1 comparing IC50s for BIRD-2 and ABT-263; each symbol represents an individual SCLC line. The horizontal line corresponds to an ABT-263 IC50 of 1 *μ*M. (**h**) H2171 cells were treated for 48 h with variable concentrations of BIRD-2, ABT-263, or 127 : 1 BIRD-2+ABT-263 (BIRD-2 IC50 to ABT-263 IC50 ratio of 127 : 1). Cell viability was measured by CTG assay

To detect BIRD-2-induced cell death, 15 SCLC lines were treated with a range of concentrations of BIRD-2 in parallel with a scrambled version of BIRD-2 (Scr) to control for non-specific peptide-mediated toxicity. After 48 h, cell viability was measured using the CellTiter-Glo (CTG) assay. Equivalent results were obtained using the AlamarBlue viability assay (data not shown). Based on these findings, IC50 values were determined (Figure 1a). BIRD-2 IC50 values were at least two-fold lower than Scr IC50 values in all SCLC lines except DMS454 and H1688, indicating that all but two of the SCLC lines are sensitive to BIRD-2-induced cell death. The sensitivity of individual lines varied considerably, as illustrated in Figures 1b and c. Among the more sensitive lines, H2171 and H1092, decreased viability was detected within 8 h following BIRD-2 addition (Figures 1d and e). Also, BIRD-2 induced cell death only in SCLC cells, showing little activity against either NSCLC or normal lung epithelial cells (Figure 1f).

The same SCLC lines were also treated with ABT-263. IC50 values for ABT-263 and BIRD-2 are detailed in Supplementary Table S1 and summarized graphically in Figure 1g. The IC50 values reflect a range of sensitivities to BIRD-2 and ABT-263, without a correlation between IC50s for BIRD-2 and ABT-263 (correlation coefficient 0.11). ABT-263 resistance generally is defined as an IC50>1 *μ*M, indicated by the horizontal line in Figure 1g.^19, 33^ According to this definition, 6 of the 15 SCLC lines were sensitive to ABT-263. If we define BIRD-2 sensitivity as BIRD-2 IC50 values at least two-fold lower than Scr IC50 values, BIRD-2 induces cell death in six of the nine ABT-263-resistant SCLC cell lines (H2029, H378, DMS79, H446, H82, H526).

In summary, the preceding findings provide the first indication that targeting Bcl-2–IP_3_R interaction, as we do with BIRD-2, can induce cell death in SCLC lines. Moreover, the findings suggest that, if therapeutic agents mimicking BIRD-2's action could be developed in the future, they might be of value in treating BH3-mimetic SCLC.

**Figure 2:**
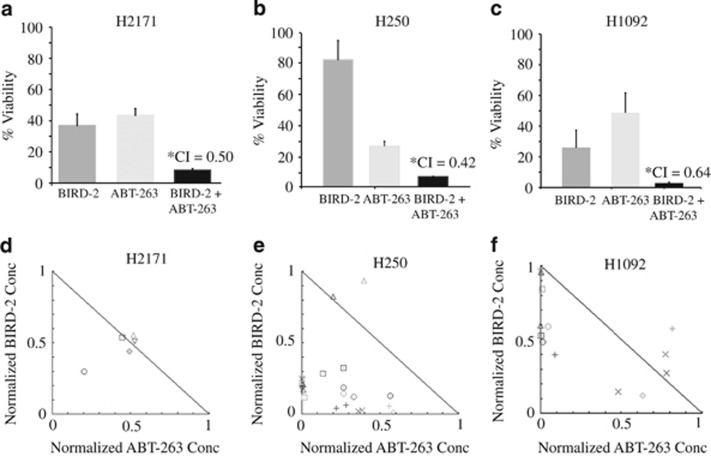
BIRD-2/ABT-263 synergy. (**a**–**c**) SCLC lines were treated with BIRD-2, ABT-263, or 127 : 1 BIRD-2+ABT-263 as in Figure 1g, with cell viability determined by CTG assay. Error bars represent mean±S.D.; *N*≥3 experiments. Asterisk designates Chou–Talalay CI calculated using the CompuSyn software, where CI<1 indicates synergy. (**d**–**f**) Normalized isobolograms for BIRD-2+ABT-263 combinations with plots generated using the CompuSyn software. Each symbol represents a unique combination of a given concentration of ABT-263 and a given concentration of BIRD-2. Symbols below the line indicate synergistic cytotoxicity; symbols on the lines indicate additive cytotoxicity; symbols above the lines indicate antagonism

Given that BIRD-2 and BH3-mimetic agents target different mechanisms by which Bcl-2 inhibits apoptosis, we tested whether combining these agents would synergistically induce cell death in SCLC cells. Consistent with this hypothesis, submaximal doses of BIRD-2 and ABT-263 significantly increased cell death in H2171 SCLC cells (Figure 1h). To establish synergy between BIRD-2 and ABT-263, cells were treated with a constant ABT-263 to BIRD-2 ratio over an entire dose–response range. This enabled mathematical assessment of synergistic *versus* additive cell killing using the CompuSyn software to calculate Combination Index (CI) values.^34^ Using a CI value of <1 as indicative of synergy, co-treatment of SCLC cells with BIRD-2 and ABT-263 consistently induced synergistic cytotoxicity in 5 of the 15 SCLC lines: H2171, H250, H1092, H526, and H1048. Three representative experiments are summarized in Figures 2a–c where bar graphs document synergistic loss of viability. Synergy is also illustrated with the use of isobolograms (Figures 2d–f), in which points below the lines indicate synergistic cytotoxicity, points on the lines indicate additive cytotoxicity, and points above the lines indicate antagonism. Notably, synergy with BIRD-2 and ABT-263 was still present at high drug effect levels, emphasizing the potential therapeutic relevance of this combination.^34^

Overall, these findings indicate that BIRD-2 is cytotoxic to a large proportion of SCLC cells tested here. Moreover, the findings indicate synergistic activity of BIRD-2 in combination with ABT-263. Finally, BIRD-2-induced cell death appears selective for SCLC, as NSCLC lines are much less sensitive to BIRD-2-induced cell death. The latter observation is consistent with expression data indicating that SCLC has higher levels of Bcl-2 than NSCLC (Supplementary Figure S1).

### Ca^2+^-mediated apoptosis in BIRD-2-treated SCLC

**Figure 3:**
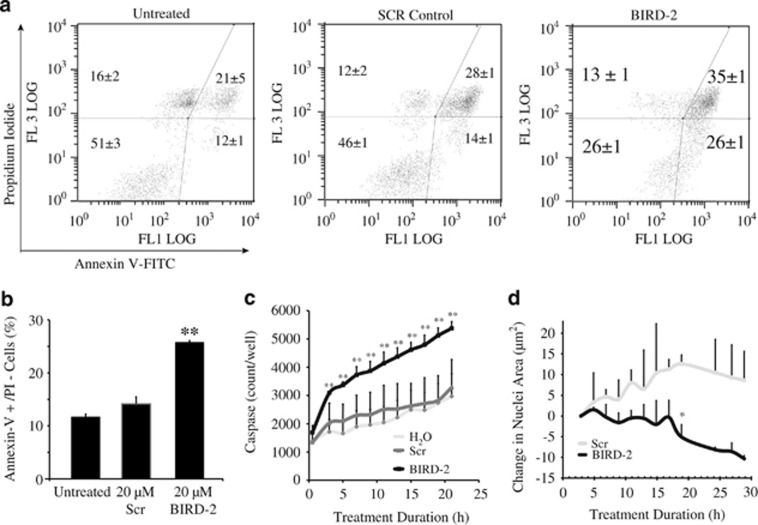
Biochemical evidence of apoptosis induction by BIRD-2. (**a**) Flow cytometry of Annexin V and propidium iodide–stained H2171 SCLC cells following treatment with 20 *μ*M BIRD-2 or Scr for 1 h. (**b**) Bar graphs (mean±S.D., three experiments) quantifying the percentage of cells, treated, and analyzed as in panel (**a**), which are Annexin V positive but propidium iodide negative. (**c**) Quantification of caspase 3/7 activity in H2171-RFP cells by IncuCyte ZOOM at various time points following addition of 20 *μ*M BIRD-2 or Scr. (**d**) Measurements of nuclear area in H2171-RFP cells treated as in panel (**c**). Error bars represent mean±S.D., *N*≥3 experiments. *P<0.05. **P<0.01

**Figure 4:**
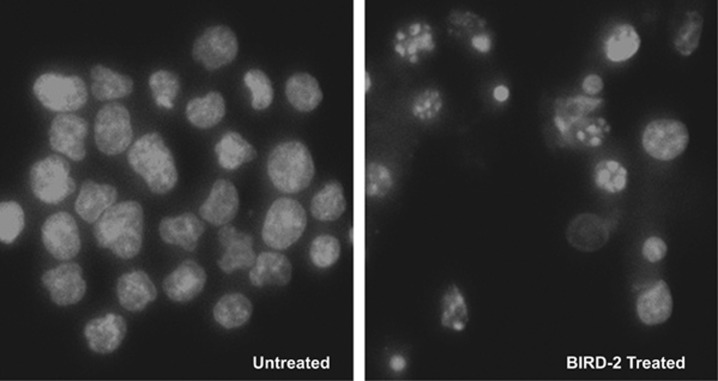
Morphological evidence of apoptosis induction by BIRD-2. H2171 SCLC cells were untreated (left) or treated for 48 h with 20 *μ*M BIRD-2 (right). Nuclei were then stained with Hoechst 33342, which fluoresces when bound to dsDNA. The nuclei of Scr-treated cells display a normal heterochromatin pattern, while the nuclei of many BIRD-2-treated cells display chromatin condensation and apoptotic bodies typical of cells undergoing apoptosis

To investigate the role of apoptosis in BIRD-2-induced cell death, we assessed several markers of apoptosis. An increase of Annexin V positivity, a marker of very early changes associated with apoptosis, was detected within 2 h of adding BIRD-2 to H2171 SCLC cells, compared with untreated and control Scr peptide-treated cells (Figures 3a and b). Note that non-viable propidium iodide-positive cells were detected in untreated and Scr-treated cells (Figures 3a and b). This is likely due to the requirement of vigorous pipetting to disrupt large clumps of SCLC cells found in culture. Because of the tendency of SCLC cells to form clumps, we turned to the use of IncuCyte ZOOM live cell imaging fluorescence microscopy. This technique analyzes cell viability and other cellular characteristics in a controlled environment tissue culture chamber without a need to disrupt cellular clumps. Red fluorescent protein (RFP)-expressing SCLC cells were used in this method to quantify caspase 3/7 activity (Figure 3c) and nuclear condensation (Figure 3d) at 2-h intervals over prolonged periods of time following addition of BIRD-2 or Scr. An example of the caspase 3/7 activity assay is included in Supplementary Figure S2. BIRD-2 treatment increased caspase 3/7 activity compared with that observed with Scr treatment in H2171-RFP cells (Figure 3c), with results reaching statistical significance within 2–3 h of treatment (*P*<0.05). BIRD-2 also induced time-dependent decreases in nuclear size in H2171-RFP cells (Figure 3d). Both of these findings are consistent with apoptosis induction. To provide more definitive morphological evidence of apoptosis, we examined Hoechst 33342-stained nuclei by fluorescence microscopy, detecting increased numbers of typical apoptotic nuclei in BIRD-2-treated cells compared with Scr peptide-treated cells (Figure 4). These findings indicate involvement of apoptosis in BIRD-2-induced cell death.

**Figure 5:**
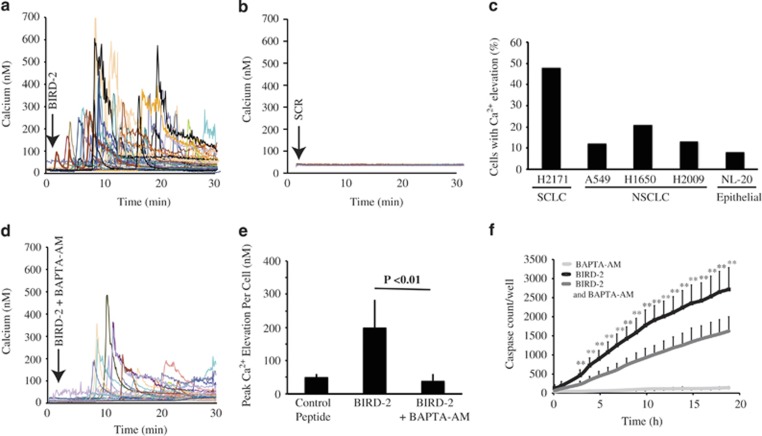
BIRD-2-induced Ca^2+^ elevation and Ca^2+^-mediated caspase activation. (**a**) Shown are continuous single-cell recordings of cytoplasmic Ca^2+^ by digital imaging, performed simultaneously in 65 H2171 SCLC cells following addition of 10 *μ*M BIRD-2 (arrow). Traces represent Ca^2+^ levels in individual cells. (**b**) Same as panel (**a**) except in Scr-treated H2171cells. (**c**) Bar graph represents the percentage of SCLC, NSCLC, and normal lung epithelial cell lines responding to BIRD-2 with a significant elevation of cytoplasmic Ca^2+^ above basal Ca^2+^ levels. Cytoplasmic Ca^2+^ was measured as in panel (**a**). This figure summarizes a single experiment analyzing >80 cells in each cell line; shown is the average peak cytoplasmic Ca^2+^ elevation in each cell line within 30 min following addition of 5 *μ*M BIRD-2. (**d**) Cytoplasmic Ca^2+^ was recorded as in panel (**a**) except that H2171 cells were pretreated 1 h before adding 10 *μ*M BIRD-2 with 5 *μ*M BAPTA-AM to chelate intracellular Ca^2+^ and thus block cytoplasmic Ca^2+^ elevation. (**e**) Shown is a bar graph representing the peak cytoplasmic Ca^2+^ elevation (mean±S.D., three experiments) induced by 10 *μ*M BIRD-2 treatment, with or without 10 *μ*M BAPTA-AM pretreatment. Results indicate that BAPTA-AM pretreatment inhibits BIRD-2-induced Ca^2+^ elevation. (**f**) BAPTA-AM pretreatment decreases BIRD-2-mediated caspase 3/7 induction, measured by IncuCyteZOOM as in Figure 3c. Experiments in panels **d**–**f** were performed at least three times, using 2–5 μM BAPTA-AM and 15–20 μM BIRD-2. Error bars in panel (**e**) represent mean±S.D., *N*=85 cells. **P<0.01

Our earlier work indicates that BIRD-2-mediated disruption of Bcl-2–IP_3_R interaction induces marked, lethal elevation of cytoplasmic Ca^2+^ concentration in Bcl-2-positive lymphoid malignancies.^26, 29, 32, 35^ BIRD-2 similarly induces a striking elevation of cytoplasmic Ca^2+^ in SCLC cells (Figure 5a), which is not observed in cells treated with Scr control peptide (Figure 5b). Ca^2+^ responses to BIRD-2 treatment were also much greater in SCLC cells than in NSCLC cells and normal epithelial cells (Figure 5c), consistent with the observation that BIRD-2 has less cytotoxic activity in these cells compared with SCLC cells (Figure 1f).

Pretreating cells with the intracellular Ca^2+^ chelator BAPTA-AM suppresses BIRD-2-induced Ca^2+^ elevation (Figure 5d), significantly decreasing BIRD-2-induced caspase activation (Figure 5e).

These findings suggest that BIRD-2 induces Ca^2+^-mediated apoptosis, as previously reported in lymphoid malignancies.^29, 31, 32^ However, although we find BIRD-2 increases caspase activity in SCLC cells, the caspase inhibitors Z-VAD-fmk and Q-VD-OH did not suppress BIRD-2-induced cell death as measured by CTG assay or BIRD-2-induced apoptosis as measured by Hoechst-stained nuclear morphology (Supplementary Figure S3). These findings suggest that BIRD-2-induced Ca^2+^ elevation may induce apoptosis by a different mechanism in SCLC cells than in lymphoid cells.

**Figure 6:**
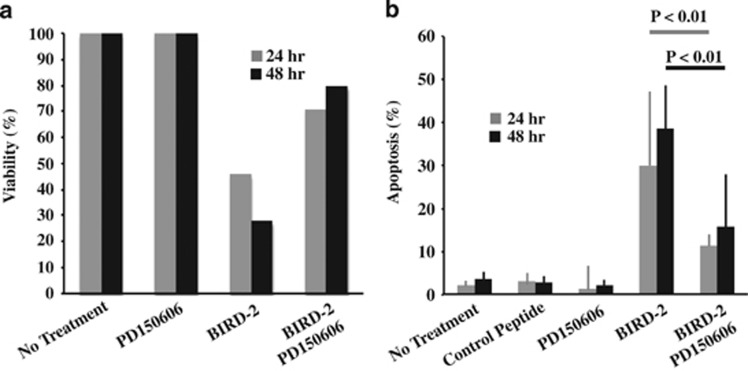
Effect of calpain inhibition on BIRD-2-induced cell death. (**a**) Bar graphs represent the percentage of viable H2171 cells, measured by CTG assay, at 24 and 48 h after various treatments as designated. Concentrations of BIRD-2 and the calpain inhibitor PD150606 were each 20 *μ*M. Results represent the mean of triplicate determinations in a single experiment and suggest that PDI150606 inhibits BIRD-2-induced cell death. (**b**) Bar graph represents the percentage of apoptotic cells, based on fluorescence microscopic detection of apoptotic nuclear morphology in H2171 cells stained with Hoechst 33342. Bars represent mean±S.D. of six images; in each image, an average of 46 cells were analyzed. This experiment was repeated twice with the same result

**Figure 7:**
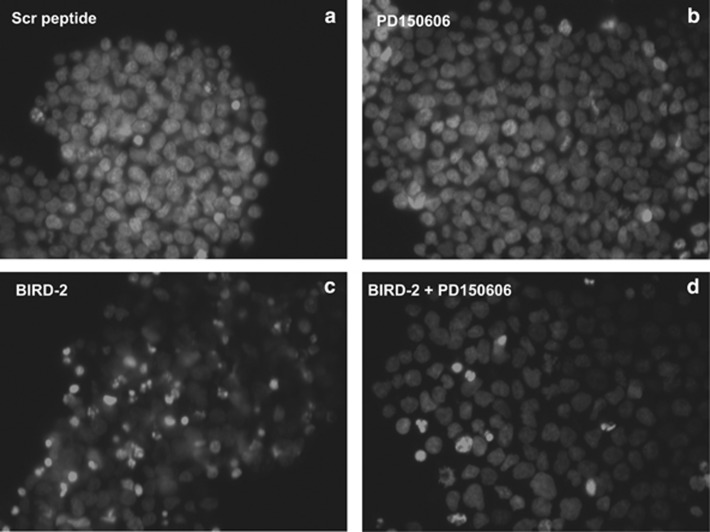
Effect of calpain inhibition on BIRD-2-induced apoptosis. Shown are representative images from Figure 6b illustrating inhibition of BIRD-2-induced apoptosis by PK150606 in Hoechst 33342-stained H2171 cells. Cells were treated for 48 h as in Figure 6 with: (**a**) 20 μM control peptide, (**b**) 20 μM PD150606, (**c**) 20 μM BIRD-2, (**d**) 20 μM BIRD-2+20 μM PD150606

Among the major known contributors to Ca^2+^-mediated apoptosis is activation of the Ca^2+^-dependent protease calpain.^36^ We find that the calpain inhibitor PD150606^37, 38^ decreases BIRD-2-induced loss of viability and apoptosis measured by the CTG assay (Figure 6a) and nuclear morphologilca changes, respectively (Figures 6b and 7). Together, these findings indicate that BIRD-2-mediated disruption of Bcl-2–IP_3_R interaction induces marked elevation of cytoplasmic Ca^2+^ in SCLC cells, triggering Ca^2+^-induced apoptosis mediated, at least in part, through calpain activation.

## Discussion

**Figure 8:**
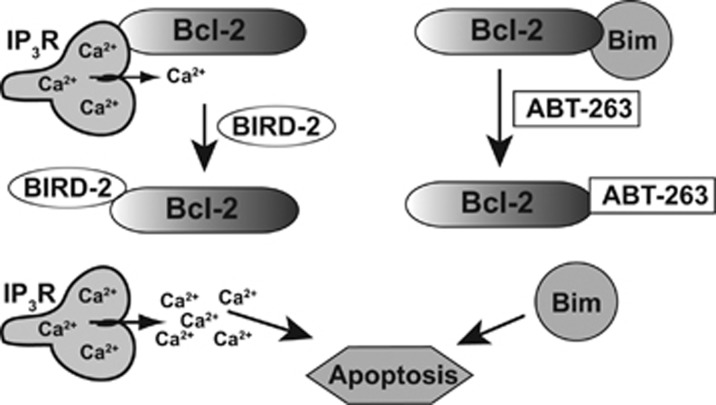
Diagram illustrating Bcl-2's dual antiapoptotic mechanisms and their differential targeting by BIRD-2 and ABT-263. Left, Bcl-2 binds to the IP_3_Rs, preventing pro-apoptotic Ca^2+^ elevation. BIRD-2 inhibits this interaction, inducing apoptosis by releasing high levels of Ca^2+^ from the ER into the cytoplasm. Right, by binding BH3-only proteins such as Bim, Bcl-2 prevents Bim from activating executioner proteins such as Bax/Bak. BH3-mimetic agents such as ABT-263 block this interaction, thereby freeing Bim to trigger apoptosis

Although Bcl-2 promotes cell survival by two fully validated mechanisms, only the mechanism involving binding and inhibition of pro-apoptotic proteins is targeted for cancer treatment by the small-molecule BH3-mimetic ABT-263 (Figure 8). To test the potential value of targeting the mechanism involving Bcl-2 interaction with IP_3_Rs in SCLC, we employed BIRD-2, a decoy peptide previously developed in our laboratory and found to induce Ca^2+^-mediated apoptosis in Bcl-2-positive lymphoid malignancies.^29, 31, 32^ We report here for the first time that BIRD-2 has single agent activity in SCLC, selectively inducing apoptosis in 13 of the 15 SCLC lines but not in NSCLC cells or normal lung epithelial cells.

For each of the 15 SCLC lines tested for BIRD-2 sensitivity, we also used the CTG assay to determine the relative sensitivity to ABT-263 (Supplementary Table S1). *In vitro* cellular sensitivity to ABT-263 has been defined as an IC50 level of ≤1 *μ*M.^19^ Following these criteria, nine of the SCLC lines tested are resistant to ABT-263. BIRD-2 induces cell death in six of these ABT-263-resistant lines (Supplementary Table S1). The demonstrated single-agent activity of BIRD-2 in ABT-263-resistant SCLC cells further supports the value of developing novel therapeutic agents that function similar to BIRD-2 by disrupting the Bcl-2–IP_3_R interaction.

BIRD-2 and ABT-263 target distinctly different regions of the Bcl-2 protein, thus inducing cell death by distinctly different mechanisms. BIRD-2 binds directly to the C-terminal BH4 domain of Bcl-2,^29, 30, 39^ whereas BH3-mimetic agents bind to a hydrophobic region of Bcl-2 composed of BH domains 1–3.^13, 14, 15^ BH3-mimetic agents induce apoptosis by displacing pro-apoptotic BH3-only proteins (e.g., Bim) from Bcl-2,^13, 14, 15^ whereas BIRD-2 does not alter Bcl-2's binding of these pro-apoptotic proteins.^28^ Conversely, BIRD-2 induces Ca^2+^-mediated apoptosis, whereas we have previously shown that ABT-263 does not alter Bcl-2 interaction with IP_3_Rs and does not induce Ca^2+^ elevation.^29^ Ultimately, these targeting approaches work in parallel to induce apoptosis by reversing separate Bcl-2-mediated pro-survival mechanisms. Thus combinations of BIRD-2 and ABT-263, as shown here, are often synergistic rather than additive. As synergy between the two compounds was most evident in 6 of the 15 SCLC lines tested, additional yet-to-be-determined factors may influence the efficacy of the ABT-263 and BIRD-2 combination.

The nature of Bcl-2's control over intracellular Ca^2+^ has been the subject of extensive investigation and is important for predicting how disruption of Bcl-2–IP_3_R interaction may potentially affect normal cell function. BIRD-2 does not kill various types of normal cells, including normal human peripheral blood lymphocytes,^29^ normal mouse embryonic fibroblasts (F Zhong and C Distelhorst, unpublished), and normal lung epithelial cells (Figure 1f). Moreover, Bcl-2 does not inhibit normal physiological Ca^2+^ signals but selectively represses high-amplitude Ca^2+^ elevations capable of inducing apoptosis.^26, 39^ We speculate that the latter property of Bcl-2 is key to exploitation of Bcl-2 by cancer cells, as Bcl-2 would allow Ca^2+^-mediated growth signals while blocking pro-apoptotic Ca^2+^ elevation, thereby promoting cancer cell survival.

In recent studies in Bcl-2-positive lymphoid malignancies, we found that the caspase inhibitor Z-VAD-fmk inhibits BIRD-2-induced apoptosis.^31^ In the present work, however, two caspase inhibitors, Z-VAD-fmk and q-VD-OH, did not inhibit BIRD-2-induced death of SCLC cells. Apoptosis, including Ca^2+^-induced apoptosis, is known to occur by both capspase-dependent and -independent mechanisms.^36^ In addition, Ca^2+^ elevation activates a variety of targets involved in mediating Ca^2+^-induced cell death.^36^ Ca^2+^-induced apoptosis in certain cell types is mediated by members of the calpain family of Ca^2+^-activated cysteine proteases.^36, 40^ Here we find that the calpain inhibitor PD150606 inhibits BIRD-2 induction of apoptosis in SCLC cells. Collectively, therefore, our findings in lymphoid malignancies and the solid tumor, SCLC, indicate that although BIRD-2 elevates Ca^2+^ levels in these different cell types, the signaling pathways leading to apoptosis appear to be different.

The current findings suggest SCLC cell survival depends in part on intracellular Ca^2+^ regulation by the Bcl-2–IP_3_R interaction. Interestingly, a recent bioinformatic screen to identify SCLC-repositioning hits identified Ca^2+^ signaling pathways among the top three hits.^41^ These findings, along with the findings reported here, highlight the relevance of altered Ca^2+^ homeostasis and signaling in SCLC. Moreover, there is considerable evidence that Ca^2+^ signaling and Ca^2+^ signaling checkpoints are remodeled in cancer cells, in part through altered regulation of IP_3_Rs, enhancing cell proliferation and survival.^42, 43^

BIRD-2 is effective at low-micromolar concentrations, which are achievable by peptides *in vivo*.^44^ Although unmodified peptides are viable clinical candidates, peptides have a number of undesirable characteristics, including susceptibility to protease cleavage, limited bioavailability, incomplete cellular uptake, and potential off-target effects.^44, 45^ The novel technology of peptide stapling has been shown to dramatically increase both *in vitro* and *in vivo* peptide biological activity.^46, 47^ Future directions include inserting hydrocarbon staples into BIRD-2 to support its further clinical development. Small-molecule inhibitors of the Bcl-2–IP_3_R interaction may also be sought in order to circumvent the potential shortcomings of unmodified peptides. A recent report of a small-molecule Bcl-2 BH4 domain antagonist that may kill lung cancer cells, at least in part, by inhibiting Bcl-2–IP_3_R interaction suggests the feasibility of such an approach.^48^ Ultimately, it is hoped that actual therapeutic agents can be developed based on the proof-of-principle evidence and patterned after BIRD-2.

## Materials and Methods

### Cell lines

SCLC, NL-20, and NSCLC cell lines were purchased from the American Type Culture Collection (ATCC, Manassas, VA, USA) with the exception of the H250 SCLC line, which was purchased from Sigma-Aldrich (St. Louis, MO, USA). The cell lines were acquired within the past 3 years and used at a passage number <10. These cell lines were not authenticated because of their direct purchase from ATCC and Sigma-Aldrich and low passage number. H2171, H1694, DMS454, H1048, SW1271, NL-20, H1650, H2009, and A549 cells were grown in 1 : 1 DMEM/F12 supplemented with 8% fetal bovine serum and 2 mM GlutaMax (Gibco Life Technologies, Grand Island, NY, USA). H378, DMS79, H250, H446, H82, H526, and H1688 cells were grown in RPMI-1640 medium, supplemented with 8% fetal bovine serum and 2 mM GlutaMax. H1092, H2029, and H64 cells were grown in 1 : 1 DMEM/F12 supplemented with 5% fetal bovine serum, 2 mM GlutaMax, 10 nM hydrocortisone, 0.005 mg/ml insulin, 0.01 mg/ml transferrin, 10 nM beta-estradiol, and 30 nM sodium selenite.

### Reagents

ABT-263 was purchased from Selleck Chemicals (Houston, TX, USA). CTG was from Promega Life Sciences (Madison, WI, USA). AlamarBlue, Hoechst 33342, Fura-2-AM, and Alexa Fluor 488 Annexin V solution were from ThermoFisher (Pittsburgh, PA, USA). PD150606 was from Sigma-Aldrich. Z-VAD-FMK was purchased from Enzo Life Sciences (Farmingdale, NY, USA) and q-VD-OH was a generous gift of Shigemi Matsuyama at Case Western Reserve University, Cleveland, OH, USA.

### Peptides

The peptide BIRD-2 (RKKRRQRRRGGNVYTEIKCNSLLPLAAIVRV) and the scrambled peptide control, Scr (RKKRRQRRRGGDLNEVTCSLIVDRINPVKLY), were synthesized by GenScript (Piscataway, NJ, USA). These peptides are described in detail elsewhere.^29^ In these peptides, the sequence RKKRRQRRRGG corresponds to the cell-penetrating peptide of HIV TAT, used to mediate peptide uptake into cells. Peptides were purified by liquid chromatography/mass spectrometry to >95% purity and were quantified by amino-acid analysis.

### Cell viability

Cell lines were cultured in T75 flasks to approximately ~50% confluency. Adherent cell lines were then trypsinized. Cells were suspended in fresh growth media, plated in 96-well plates at 10 000 cells/well, and allowed to settle overnight at 37 °C. Cells were then treated for various periods of time and with various concentrations (0.4–200 *μ*M) of either BIRD-2 or Scr, as stated under Results section. Viability was determined using either the CTG or AlamarBlue viability assays. Dose–response curves were generated in Microsoft Excel (Microsoft, Redmond, WA, USA); cell-death IC50 values were calculated using the GraphPad Prism Statistical Software (La Jolla, CA, USA). Experiments were performed in triplicate; error bars represent S.D.

For CTG viability experiments, SCLC cells were added to 96-well plates and treated as above. At time points designated under Results section, CTG assay reagent (Promega) was diluted five-fold in PBS, and 80 *μ*l of the diluted reagent was added to each well. Plates were agitated for 2 min and then incubated at rest for 10 min. Total luminescence of each well, corresponding to ATP content, was recorded using a VICTOR3 Microplate reader (PerkinElmer, Akron, OH, USA).

To confirm the validity of the CTG assay as a surrogate for cell viability in SCLC cells, the AlamarBlue viability assay was performed in parallel in a number of experiments. For the AlamarBlue assay, SCLC cells were added to 96-well plates and treated as above. Eight *μ*L of AlamarBlue reagent (Life Technologies, Grand Island, NY, USA) was added to each well, mixed thoroughly, and allowed to incubate at 37 °C for 2–4 h. Fluorescence values of each well, corresponding to the reductive capacity of the cell, were generated using an Envision-2103 Microplate reader (PerkinElmer) with an excitation filter of 560 nm and an emission filter of 590 nm.

### Synergy determination

Following the Chou–Talalay method,^34^ IC50 values for BIRD-2 and ABT-263 were first assessed in SCLC cell lines by CTG assay. Ten thousand SCLC cells were then added to each well of a 96-well plate and were treated for 48 h with serial dilutions of BIRD-2 and ABT-263. In the H2171 cell line, BIRD-2 and ABT-263 were combined at maximum doses equal to four times their individual IC50s and then serially diluted at a constant BIRD-2:ABT-263 ratio. Serial dilutions of ABT-263, BIRD-2, Scr, and H_2_O alone were included as controls. Viability data were generated using the CTG assay as described above. CI values were generated using the CompuSyn software; synergy was defined as a CI<1, according to Chou–Talalay method.^34^

### Flow cytometry

Flow cytometry was used to measure apoptosis according to externalization of phosphatidylserine, detected by Annexin V, and to simultaneously measure increased membrane permeability, detected by propidium iodide uptake. Five hundred thousand SCLC cells were added to 35-mm dishes and treated for 1 h with BIRD-2 or Scr. Cells were then harvested, washed in PBS, and stained for 15 min in the dark with a 30-fold dilution of Alexa Fluor 488 Annexin V solution and 1 *μ*g/ml propidium iodide. Stained samples were diluted 5 × in annexin-binding buffer. Samples were analyzed in the Case Comprehensive Cancer Center Cytometry Core Facility on an EPICS-XL flow cytometer (Beckman Coulter, Pasadena, CA, USA). Data analysis was performed using WinList (Verity House Software, Topsham, ME, USA).

### IncuCyte ZOOM

IncuCyte ZOOM (Essen Biosciences, Ann Arbor, MI, USA) was used to detect caspase activation and nuclear condensation as described by others.^49^ The CellPlayer NucLight Red lentiviral construct (Essen Biosciences) was inserted into H2171, SW1271, and H1092 SCLC cells (hereafter referred to as H2171-RFP, SW1271-RFP, and H1092-RFP cells), using their standard infection protocol. The H2171-RFP, SW1271-RFP, and H1092-RFP cell lines were maintained in 0.5 *μ*g/ml puromycin to select for RFP-infected cells. Successful insertion of the NucLight Red lentiviral construct was confirmed via direct visualization of transfected cells on the IncuCyte ZOOM fluorescent microscope. For IncuCyte ZOOM experiments, H2171-RFP, SW1271-RFP, and/or H1092-RFP SCLC cells were suspended in fresh growth media, plated in 96-well plates at 10 000 cells/well, and allowed to settle overnight at 37 °C. Caspase 3/7 activity was assessed using the Essen Biosciences IncuCyte ZOOM caspase 3/7 assay, as described previously.^49^ Briefly, the CellPlayer 96-Well Kinetic Caspase 3/7 Reagent (designated hereafter as caspase 3/7 reagent) consists of an inert peptide, a caspase 3/7 recognition site, and the peptide NucView 488 (Essen Biosciences). The full-length caspase 3/7 reagent is non-fluorescent and is confined to the cytoplasm. Upon induction of apoptosis, caspase 3/7 cleaves the bond between the inert peptide and NucView 488. Liberated NucView 488 has a high affinity for nuclear DNA and is fluorescent in the green spectrum; thus caspase 3/7 activation correlates with an increase in fluorescent green nuclei. To assess nuclear condensation, SCLC cells were cultured in 96-well plates and monitored in the IncuCyte ZOOM acquiring images every 2 h following treatment with ABT-263, BIRD-2, or Scr control peptide. Average areas of RFP-labeled nuclei were determined at each time point using the IncuCyte ZOOM analysis software.

### Apoptotic morphology

Apoptotic nuclear morphology was evaluated in wild-type (i.e., RFP-negative) SCLC cells stained with Hoechst 33342 (10 *μ*g/ml) for 15 min at 37 °C and visualized by epifluorescence microscopy (Photo Technology International EasyRatio Pro imaging platform, Photon Technology International, London, Ontario, Canada) equipped with dual excitation and emission capability and excitation wavelength selection by monochromater. Images were recorded using a Zeiss Axio ObservervA1 inverted microscope with × 40 fluor oil objective (Carl Zeiss Microscopy, Thornwood, NY, USA) and Roper CoolSnap digital camera (Photometrics, Tucson, AX, USA).

### Calcium measurement

Techniques for single-cell digital imaging of Fura-2-AM-loaded cells are described previously.^29^

### Statistical methods

Single-agent caspase 3/7 experiments were performed in octuplicate (4 images/well × 2 wells); error bars correspond to 1 S.D. The *P*-values were generated via the GraphPad Prism statistical software, using unpaired *t*-tests to compare H_2_O *versus* BIRD-2, H_2_0 *versus* Scr, and BIRD-2 *versus* Scr. Dual-agent caspase 3/7 experiments were performed in 48-plicate (4 images/well × 12 wells); error bars correspond to S.D. Unpaired *t*-tests compared treatment with BIRD-2 and ABT-263 *versus* BIRD-2 alone. All other biochemical and cellular assays had a sample size of three replicates for each comparison of experimental *versus* control conditions. This sample size determination was based on the following experimental constraints: a power of 0.80; expected S.D. of ±10% maximum value; powered to detect differences of ±25% maximum value, and type 1 error rate (*P*-value) of 0.05. Average values and S.Ds. were calculated using Microsoft Excel. The IC50 of dose–response curves was generated by using the GraphPad Prism software to fit data to a ‘nonlinear regression: log(inhibitor) *versus* response–variable slope' model. *P*-values were calculated for all experiments using th GraphPad Prism software to conduct unpaired *t*-tests between experimental and control conditions. Statistically significant differences were defined as those with *P*<0.05 between experimental and control conditions. Power and sample size estimations were calculated using the GraphPad StatMate software: http://graphpad.com/scientific-software/statmate/. For all cellular assays, 5000–10 000 SCLC cells were used per experimental condition tested.

## Abbreviations

BIRD-2: Bcl-2 IP3 receptor disruptor-2
CI: Combination Index
CLL: chronic lymphocytic leukemia
CTG: CellTiter-Glo
ER: endoplasmic reticulum
IP_3_R: inositol 1,4,5-trisphosphate receptor
NSCLC: non-small cell lung cancer
RFP: red fluorescent protein
SCLC: small cell lung cancer
Scr: scrambled BIRD-2
