# Supplementary material for: Synergistic killing of human small cell lung cancer cells by the Bcl-2-inositol 1,4,5-trisphosphate receptor disruptor BIRD-2 and the BH3-mimetic ABT-263
Source: Cell Death Dis. 2015 Dec 31;6(12):e2034–. doi: 10.1038/cddis.2015.355 (PMC4720890; doi:10.1038/cddis.2015.355)
Supplement: Supplementary Table S1 [file cddis2015355x1.pdf]

Cell Line	BIRD-2 IC50 (μM)	StDev	Scr IC50 (μM)	StDev	ABT-263 IC50 (μM)	StDev
H2171	9	3	23	2	0.05	0.03
H1092	9	2	32	5	1	0.1
H2029	9	11	33	15	1.5	1.4
H64	9	3	30	6	0.17	0.08
H378	10	3	45	3	>4	N/A
DMS79	14	8	53	11	2.4	0.6
H1694	18	7	67	1	0.11	0.02
H250	18	4	55	6	0.37	0.07
H446	20	9	78	4	1.5	0.1
DMS454	22	6	34	11	0.41	0.1
H82	23	4	57	7	>4	N/A
H526	24	2	60	0	7.8	5
H1048	33	3	75	1	2.2	0.1
SW1271	33	4	79	11	26	7
H1688	56	27	43	1	1.6	0.4

Supplementary Table 1. BIRD-2 and ABT-263 sensitivity of SCLC lines
