# Supplementary material for: Synergistic killing of human small cell lung cancer cells by the Bcl-2-inositol 1,4,5-trisphosphate receptor disruptor BIRD-2 and the BH3-mimetic ABT-263
Source: Cell Death Dis. 2015 Dec 31;6(12):e2034–. doi: 10.1038/cddis.2015.355 (PMC4720890; doi:10.1038/cddis.2015.355)
Supplement: Supplementary Figure S1 [file cddis2015355x2.pdf]

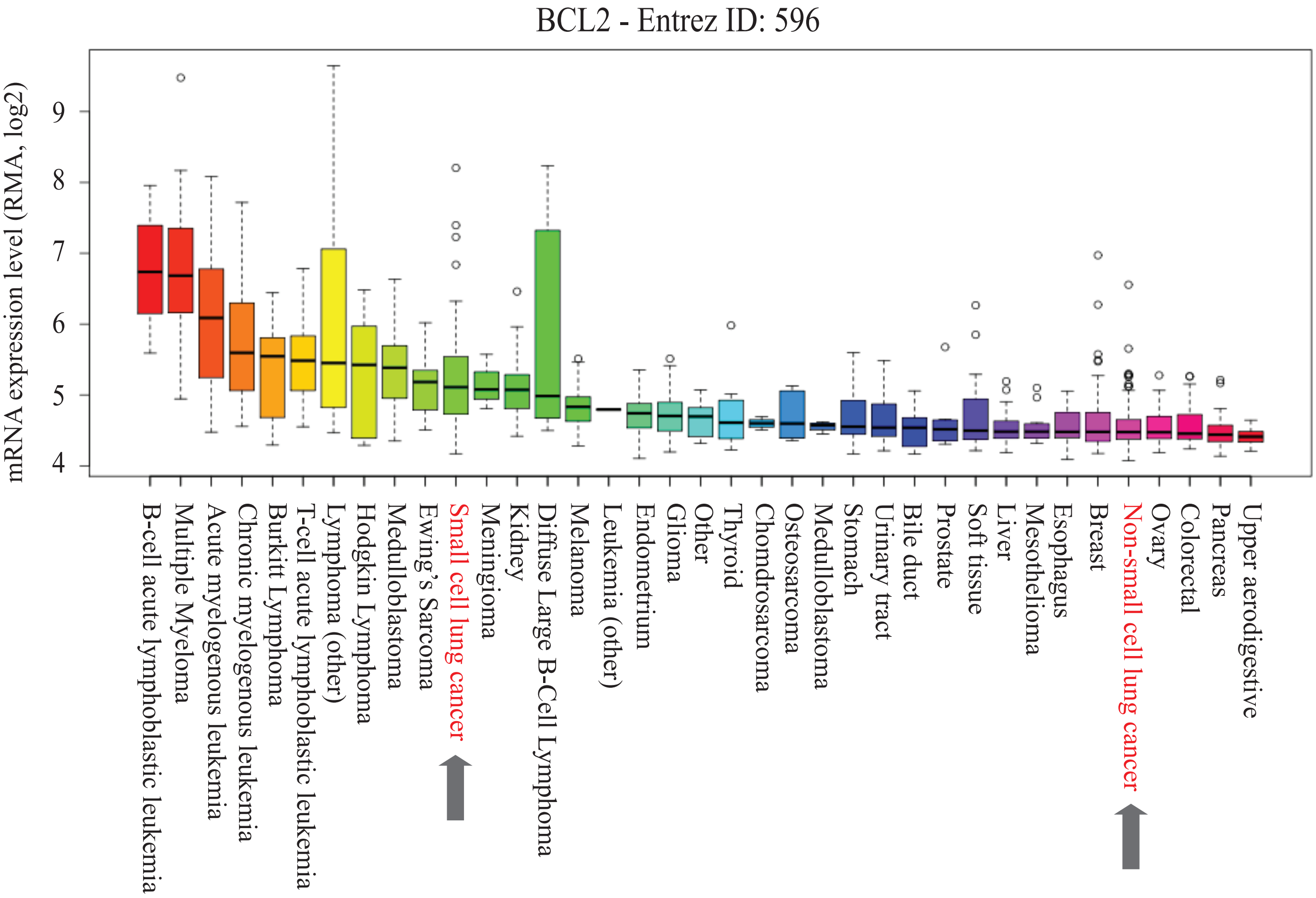

Supplementary Figure 1. Bcl-2 mRNA levels in malignancies of different cellular origin. SCLC has a higher Bcl-2 mRNA level than many other solid tumors. This figure is derived from the Broad Institute's Cancer Cell Line Encyclopedia Database: http://www.broadinstitute.org/ccle/home. *Arrows,* SCLC and NSCLC.
