# Supplementary material for: Synergistic killing of human small cell lung cancer cells by the Bcl-2-inositol 1,4,5-trisphosphate receptor disruptor BIRD-2 and the BH3-mimetic ABT-263
Source: Cell Death Dis. 2015 Dec 31;6(12):e2034–. doi: 10.1038/cddis.2015.355 (PMC4720890; doi:10.1038/cddis.2015.355)
Supplement: Supplementary Figure S2 [file cddis2015355x3.pdf]

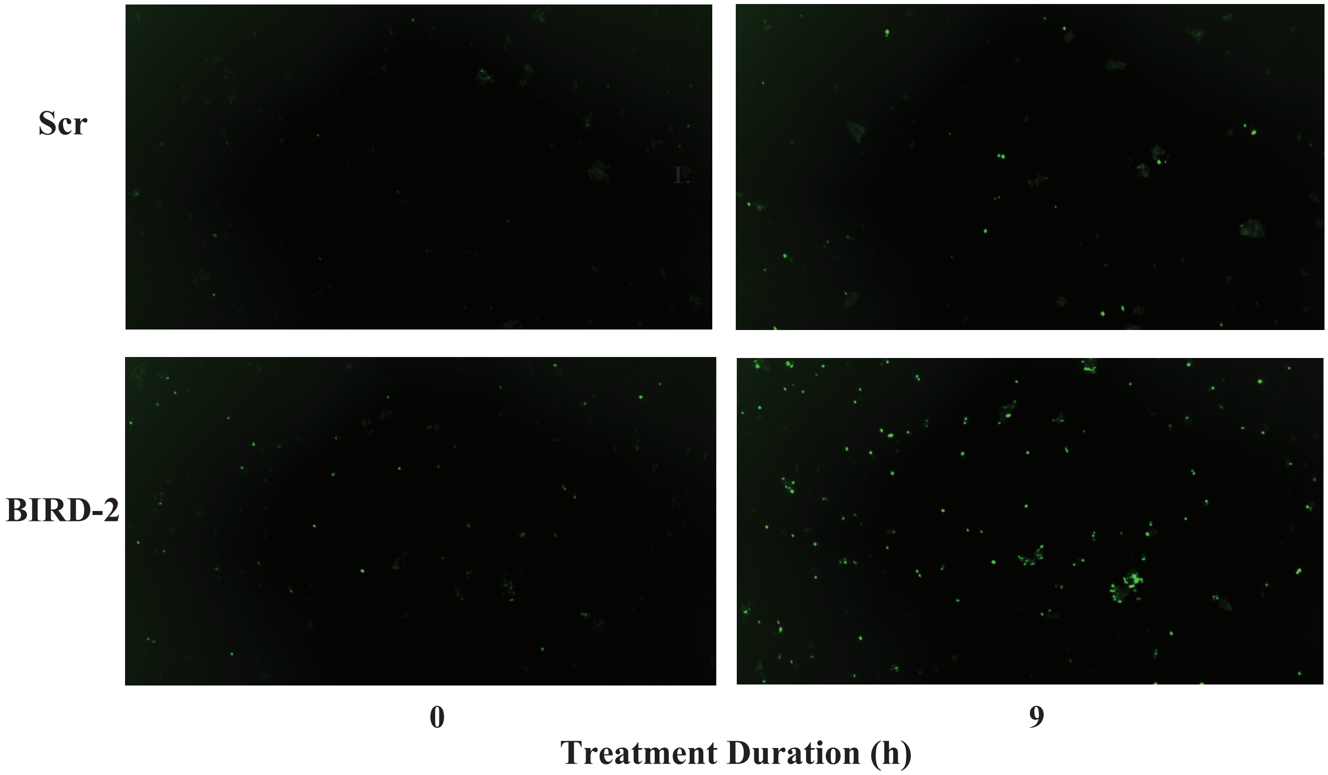

Supplementary Figure 2: Caspase 3/7 activity in SCLC Cells. Representative images obtained from the IncuCyte ZOOM, measuring caspase 3/7 activity in H2171 cells treated with 20 μM BIRD-2 or Scr control peptide for the indicated times.
