# Supplementary material for: Synergistic killing of human small cell lung cancer cells by the Bcl-2-inositol 1,4,5-trisphosphate receptor disruptor BIRD-2 and the BH3-mimetic ABT-263
Source: Cell Death Dis. 2015 Dec 31;6(12):e2034–. doi: 10.1038/cddis.2015.355 (PMC4720890; doi:10.1038/cddis.2015.355)
Supplement: Supplementary Figure S3 [file cddis2015355x4.pdf]

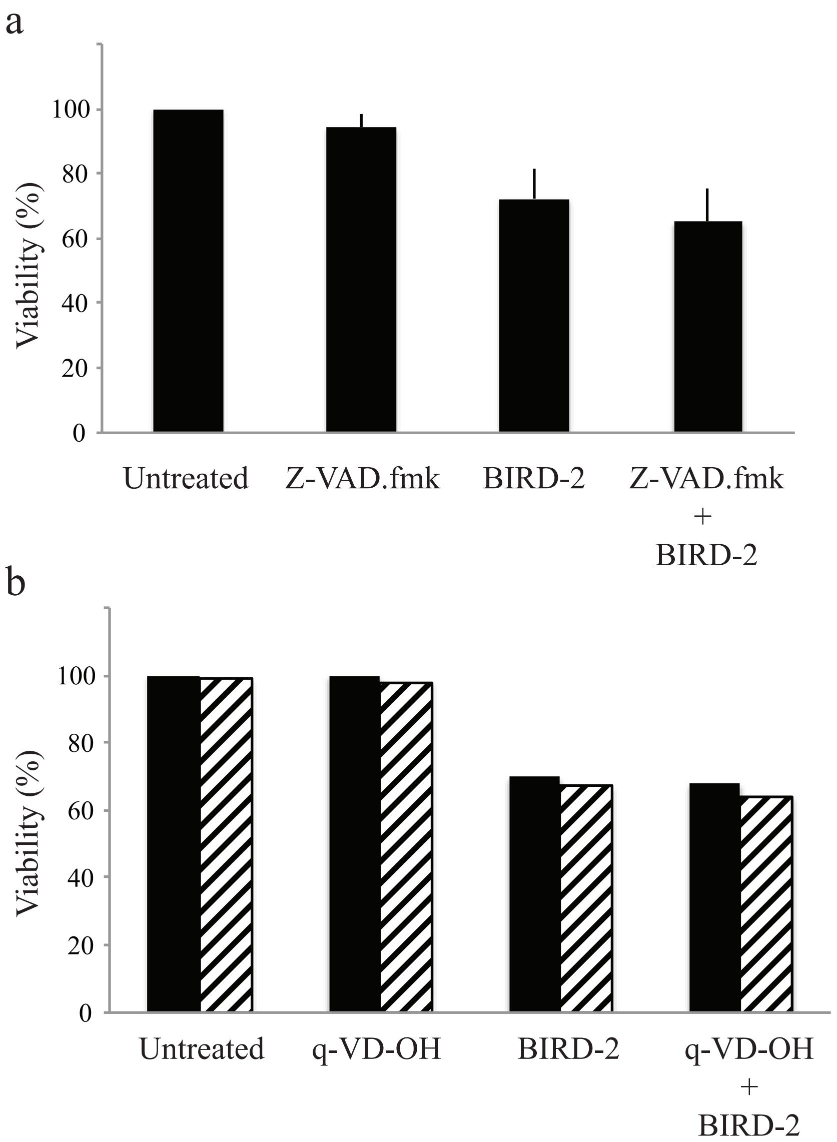

Supplementary Figure 3. Effect of caspase inhibitors on BIRD-2-induced cell death.
(a) H2171 SCLC cells were treated for 24 h with 20 µM BIRD-2 ± 200 µM Z-VAD.fmk. Cell viability was determined by CTG assay with data normalized to untreated cells. Symbols represent mean ± SD (N=4 separate experiments). (b) H2171 SCLC cells were treated for 24 h with 20 µM BIRD-2 ± 20 µM q-VD-OH. Solid bars represent findings of a single experiment in which cell vaibility was determined by CTG assay. Cross-hatched bars represent findings of a separate experiment in which cell apoptosis was detected based on morphology of Hoechst-stained nuclei, and viability measurement calculated as percentage of cells with normal nuclear chromatin pattern and absence of apoptotic hallmarks.
